# Untargeted Swab Touch Spray-Mass Spectrometry Analysis with Machine Learning for On-Site Breast Surgical Margin Assessment

**DOI:** 10.1021/acs.analchem.4c06062

**Published:** 2025-01-19

**Authors:** Laura Min Xuan Chai, Ching Kao, Ming-Yang Wang, Cheng-Chih Hsu

**Affiliations:** † Department of Chemistry, National Taiwan University, Taipei 10617, Taiwan; ‡ Department of Surgical Oncology, National Taiwan University Cancer Center, Taipei 10672, Taiwan; § Leeuwenhoek Laboratories Co. Ltd., Taipei 10672, Taiwan

## Abstract

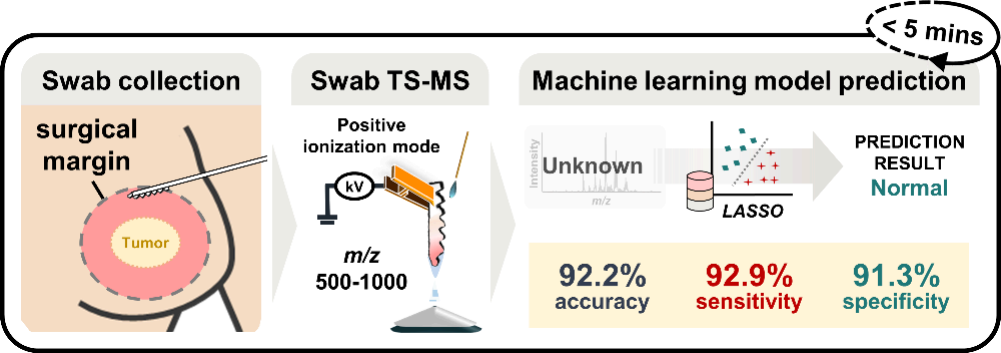

Direct sampling mass spectrometry (MS) has rapidly advanced with the development of ambient ionization MS techniques. Swab touch-spray (TS)-MS has shown promise for rapid clinical diagnostics. However, commercially available swabs are notorious for their high background signals, particularly in the positive ionization mode. Although changes to MS methods or precleaning of the swabs can serve as workarounds, this inherent issue still limits the clinical application of swab TS-MS. In this study, we report the use of the sterile-packaged OmniSwab as an alternative material for untargeted swab TS-MS analysis. As a proof of concept, breast surgical margins were swabbed *in vivo* during surgeries and analyzed using a compact mass spectrometer within the hospital. Subsequently, various machine learning algorithms were applied to the acquired MS spectra to determine the optimal model for classifying margins as normal or tumor. The Least Absolute Shrinkage and Selection Operator (LASSO) model yielded the highest prediction performance, with accuracies exceeding 90% in both testing and validation data sets. Notably, three out of four surgical margins involved with cancer cells were accurately identified. The entire workflow, from swab TS-MS analysis to margin prediction, can be completed within 5 min with high accuracy, demonstrating the feasibility of swab TS-MS to assist intraoperative decision-making.

Mass spectrometry (MS) has been extensively explored in clinical applications due to its capability for label-free multiplex detection of compounds in biological specimens at high sensitivity and selectivity. Conventional MS methods typically involve separation using chromatography, resulting in the need for sample preparation prior to MS analysis.^1^ With the development of ambient ionization mass spectrometry (AIMS) techniques, such as desorption electrospray ionization (DESI) and direct analysis in real time (DART), samples can now be introduced into MS systems with minimal or even no sample pretreatment.^2−4^ In recent decades, direct sampling MS methods have been developed to provide rapid *in situ* feedback. Techniques like MasSpec Pen,^5^ rapid evaporative ionization mass spectrometry (REIMS),^6^ liquid microjunction surface sampling probe (LMJ-SSP),^7^ and water-assisted laser desorption/ionization technology (SpiderMass)^8^ allow for real-time, *in vivo* tissue analysis. Other *ex vivo* spray-based methods include ionization of analytes deposited on substrates, such as paper spray ionization (PSI),^9^ or on probe surfaces, such as probe electrospray ionization (PESI)^10^ and touch spray mass spectrometry (TS-MS).^11^

TS-MS is appealing due to its straightforward handling and analysis of samples, as both sample collection and ionization can be performed on the same probe. It typically involves a metallic sharp tip as the sampling device, which is then wetted with a small volume of solvent, generating charged droplets from a point where applied potential is sufficiently strong.^11,12^ An extension of this technique, known as swab TS-MS, uses medical swabs as the sampling probe, making it noninvasive and more easily accessible. Unlike conventional TS-MS which generates ions from the sharp tip, ions form from the porous surface of swabs, much like PSI.^13^ Taylor cones are observed at the tip of the swab head after applying solvent and electric potential.^14^ Swab TS-MS has been demonstrated for identifying strep-throat causing bacterium,^13^ direct analysis of drugs of abuse in oral fluids,^15,16^ and determining the gene mutation status of gliomas to assist with surgical decisions,^17^ thereby highlighting its feasibility for rapid clinical testing.

Despite the potential offered by swab TS-MS, commercially available swabs are notorious for producing high background signals in positive ionization mode, particularly in higher mass range (*m*/*z* > 500), owing to the polymers and chemicals present in swabs.^13,15,18^ This phenomenon was also observed when swabs were analyzed using other AIMS techniques.^19,20^ The presence of contaminants may cause peak overlaps and ion suppression of molecules of interest during sample analysis. Hence, most swab TS-MS analyses are performed in negative ionization mode, though swab materials may still exhibit some interference peaks.^13^ When positive ionization mode is required, the possible solutions involve selecting swabs with relatively “cleaner” backgrounds,^19^ and performing multiple stages of targeted mass analysis (MS^n^) over the lower background signals.^15^ While sonicating swabs in ethyl acetate before sampling can effectively reduce interference peaks,^18^ it increases processing time, and the sterility of the swab becomes questionable. This essentially restricts the utility of swab TS-MS in clinical settings.

To expand the applicability of swab TS-MS, we sought a sterile-packaged commercial swab material that does not produce significant interference signals in positive ionization mode. We subsequently discovered OmniSwab, a swab made of compressed cellulose fiber used for collecting buccal cell DNA,^21^ which shows great potential for the above-mentioned purpose. Breast cancer surgeries present an excellent application for swab TS-MS. Determining negative margins around the excised tumor is crucial for reducing the risk of cancer recurrence.^22^ However, approximately 10–40% of patients undergoing breast-conserving surgeries have margins positive for tumor cells, necessitating a second operation for margin clearance.^23,24^ Intraoperative margin information could therefore help in avoiding repeat surgeries and improve patient outcomes.

Several AIMS techniques have been developed for the breast surgical margin assessment. DESI-MS imaging was the first, enabling delineation of tumor margins through their lipidomic profiles.^25^ REIMS later fulfilled the need for *in vivo* evaluation of breast tissues, in which *ex vivo* prediction models achieved a 90.9% sensitivity and 98.8% specificity in the validation set, and had high recognition rates of intraoperative spectra.^26^ A subsequent multicenter study revealed similar spectral information across three study sites, supporting the development of a highly accurate global REIMS-based classification model.^27^ While *in vivo* analysis using LMJ-SSP was also possible, its classification ability has yet to been validated.^28^ The MasSpec Pen has also shown promise for intraoperative breast tissue evaluation, with 147 analyses from 22 cases showing 95.9% agreement with pathological results.^29^

Building upon these advances, we present the first demonstration of swab TS-MS coupled to a compact mass spectrometer for analyzing *in vivo* breast surgical margins. While LC-MS^30,31^ and other AIMS techniques^5,32,33^ have frequently employed untargeted profiling and machine learning (ML) to facilitate accurate disease diagnosis, this approach remains unexplored for swab TS-MS. Therefore, our study also investigated the applicability of untargeted mass profiles from swab TS-MS in positive ionization mode in combination with ML to assist in intraoperative decision-making. The workflow is depicted in Figure 1.

**Figure 1:**
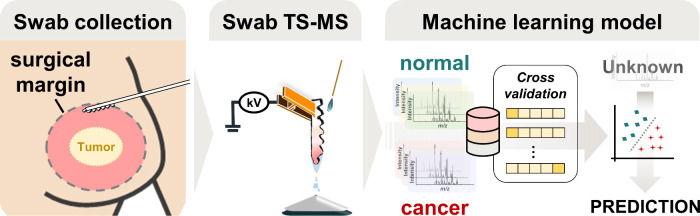
Schematic of swab collection and prediction model construction using untargeted swab TS-MS profiles and machine learning.

## Experimental Section

### Swab Collection

Samples were acquired from breast-conserving surgeries at the National Taiwan University Cancer Center (NTUCC). The study was approved by the Institutional Review Board and Ethics Committee of the National Taiwan University Hospital (IRB approval no. 202108123RINA). Patient demographics are detailed in Supporting Information (SI) (Table S1). OmniSwab (Qiagen, Germany) was used to swab *in vivo* surgical margins in which its clock-face orientation was noted. To acquire cancerous tissue for ML model construction, an area including both tumor and normal tissue was swabbed. Additional swabs were also obtained during breast mastectomy surgeries. Swabs were then delivered to a clinical examination room at NTUCC within 20 min for swab TS-MS analysis. After surgery, tissue pathology report was referred to confirm if the swabbed tumor is cancerous, and that sampled surgical margins are free of cancer cells. Swabs of negative surgical margins are labeled as normal breast tissue, while those of positive surgical margins are labeled as margin-involved.

### Swab TS-MS Analysis and Machine Learning Model Construction

**Figure 2:**
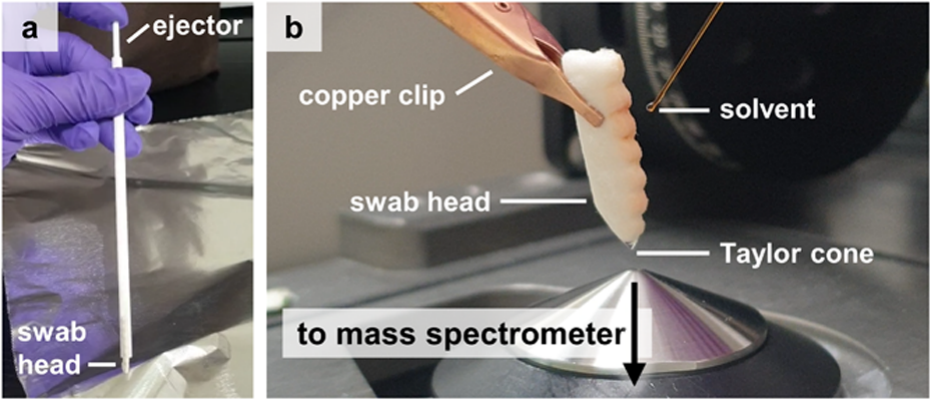
(a) OmniSwab, which has an ejectable swab head. (b) Swab TS-MS experimental setup. High voltage is applied via the copper clip, while continuous solvent supply is delivered through a syringe pump (not pictured).

Swab head of OmniSwab can be detached from the handle by pushing the ejector (Figure 2a). The swab TS-MS experimental setup on the compact mass spectrometer, Advion Expression^L^ CMS, is shown in Figure 2b (whole mass spectrometer not pictured). The instrument measures 66 × 28 × 56 cm^3^ (H × W × D) and weighs 30 kg, making it easily adaptable to available spaces.

Briefly, the ejected head is clipped at the opposite edge of the swabbing end using a copper clip, where a +4 kV voltage was applied. The distance between the swab tip and mass spectrometer orifice was ∼6 mm and at a ∼70–90° angle to facilitate Taylor cone formation. 250 μL of 50:50 (v/v) water:methanol was added to wet the swab. A mixture (50:35:15, v/v/v) of methanol, acetonitrile, and ethyl acetate was then used to extract the compounds from the swab at a flow rate of 25 μL/min, with the solvent directed toward the top corner of the swabbing edge or the most tissue-swabbed area. The continuous wicking helps to maintain the Taylor cone at the suspended droplet on the swab tip. The capillary temperature was set at 250 °C. Approximately 20 s after stable Taylor cone formation, spectra were collected from *m*/*z* 100–1000 for 0.3 min to compare between various swab materials (details in Supporting Information). All swabs were analyzed in the same manner as that for OmniSwab. Since the handles of other swabs were not ejectable, the copper clip was attached at the point where the swab head and handle intersect. For breast surgical margin assessment, a 0.3 min spectrum was acquired from *m*/*z* 500–1000.

Prior to ML model construction, spectra were averaged, normalized to the total ion count (TIC), and binned into 1 Da intervals after *m*/*z* alignment. Three ML algorithms from the sklearn package were evaluated for their tissue classification performance, with feature selection applied: Least Absolute Shrinkage and Selection Operator (LASSO), Support Vector Machine with Recursive Feature Elimination (SVM-RFE), and Random Forest with Boruta (RF-Boruta). Prediction performance was evaluated using the sensitivity, specificity and accuracy. Surgeons were blinded to swab TS-MS prediction results throughout the study.

## Results and Discussion

### Swab Material Comparison

Various swabs were tested as probes for swab TS-MS. The selected swab should not only produce low background signals in positive ionization mode but also be capable of analyzing masses across a range of *m*/*z* values. Hence, their profiles were compared using an ESI tuning mix, which consisted of standards at *m*/*z* 118.09, *m*/*z* 322.05, *m*/*z* 622.03, and *m*/*z* 922.01. The test was conducted as described in the procedures, with the wetting and extraction solvent replaced by an ESI tuning mix. A direct infusion electrospray ionization (ESI) profile was also obtained at a flow rate of 30 μL/min for reference. Their profiles over the *m*/*z* range of 100–1000 are depicted in SI, Figure S1, where OmniSwab (SI, Figure S1b) had the least spectral background. Other swab materials (SI, Figure S1c–g) exhibited polymer-like peaks, mostly starting from *m*/*z* 400, leading to peak overlaps and suppressed standard peaks. Note that swabs of the same material from different manufacturers may produce different background signals, as seen with the cotton swabs in SI, Figure S1c,d. As expected, interference signals from swab material are minimal in negative ionization mode (SI, Figure S2), consistent with previous studies,^13,20^ and all standard peaks were observed.

### Swab TS-MS Analysis of Breast Tissues

**Figure 3:**
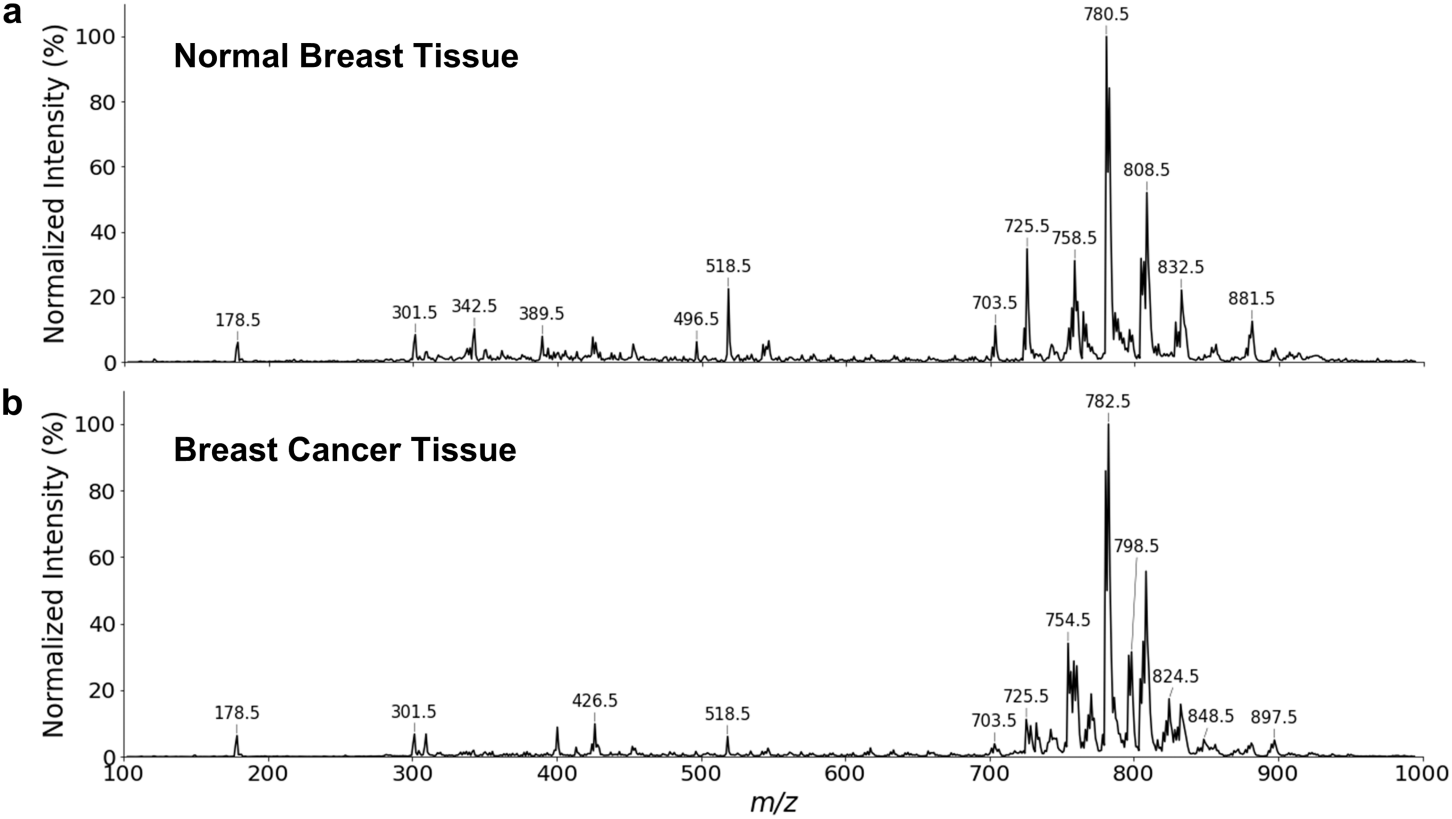
TIC normalized swab TS-MS profiles in positive ionization mode, averaged over 0.3 min and binned into 1 Da resolution, acquired from swabbing (a) normal breast tissue and (b) breast cancer tissue of the same patient.

Swab TS-MS has the potential to provide margin information to surgeons intraoperatively, thereby assisting in surgical decision-making. To explore the feasibility of this application, swabs of breast tissues were analyzed using this technique on a compact mass spectrometer. The location of each swabbed region was noted during the sample collection. This spatial information will help guide surgeons toward areas that require further tissue resection based on swab TS-MS prediction results. It also enables cross-referencing with the pathology report to confirm whether the swabbed regions were normal or cancerous. Figure 3 shows the preprocessed spectra acquired from the normal and cancerous tissue of a patient.

MS signals were most abundant in the *m*/*z* region of 700–900, which is attributed to lipids in breast tissue.^33^ Qualitatively, the relative abundance of compounds varies between different tissue types, for instance, higher abundances observed in breast cancer tissue for *m*/*z* bin 754.5 and *m*/*z* bin 798.5. Low-molecular-weighted species were also present, with minute differences in *m*/*z* of 300–500. Note that the maximum duration of swab TS-MS analysis is highly dependent on the amount of tissue deposited on the swab, with TIC signal stability ranging from 2 to 15 min (SI, Figure S3). Therefore, a 0.3 min spectrum was acquired to maximize data collectability.

### Machine Learning Model Construction for Breast Surgical Margin Assessment Using Swab TS-MS

For breast surgical margin assessment, 109 samples (51 normal tissue and 58 cancer tissue) were split at an 8:2 ratio for training and testing. Three ML algorithms, namely, LASSO, SVM-RFE, and RF-Boruta, were evaluated for their tissue classification performance. Feature selection was introduced to SVM and RF during training to reducing the risk of model overfitting and improve model interpretability.^34,35^ This was not necessary for LASSO, as regularization leads to feature selection by reducing the coefficients of less important features to zero.^33,35^ The hyperparameters of the ML models were optimized through 5-fold cross validation (CV) on the training set, and later evaluated with the testing data and an external validation set consisting of 23 normal, 24 tumor, and 4 margin-involved samples. The prediction performance of the models across the data sets is as shown in Table 1.

**Table 1:** Performance of Constructed Machine Learning Models Using Swab TS-MS Data in *m/z* Range of 500 to 1000

performance	LASSO	SVM-RFE	RF-Boruta
Training Data Average 5-Fold CV (±SD)			
accuracy	89.7% (±4.1%)	92.0% (±6.0%)	90.7% (±4.8%)
specificity	85.6% (±8.8%)	92.8% (±5.9%)	92.5% (±6.1%)
sensitivity	93.8% (±8.1%)	91.1% (±13.0%)	88.9% (±12.2%)
Testing Data (Correct Predictions/Number of Samples)			
accuracy	90.9% (20/22)	86.4% (19/22)	90.9% (20/22)
specificity	90.0% (9/10)	90.0% (9/10)	100.0% (10/10)
sensitivity	91.7% (11/12)	83.3% (10/12)	83.3% (10/12)
Validation Data (Correct Predictions/Number of Samples)			
accuracy	92.2% (47/51)	90.2% (46/51)	84.3% (43/51)
specificity	91.3% (21/23)	100.0% (23/23)	95.7% (22/23)
sensitivity	92.9% (23/24 + 3/4)	82.1% (22/24 + 1/4)	75.0% (19/24 + 2/4)

All three algorithms performed comparably well on the training data, achieving average 5-fold CV accuracies of 89.7%, 92.0%, and 90.7%, respectively. However, standard deviations exceeding 10% sensitivity were noted for SVM-RFE and RF-Boruta. While performance was similar for all models on the testing data, LASSO demonstrated the highest accuracy in the validation set as the other two models experienced decreased sensitivity. Despite SVM-RFE having a specificity of 100%, it was capable of predicting only one out of four margin-involved samples. Hence, the LASSO model was ultimately selected for its balanced performance, achieving both sensitivity and specificity above 90% and successfully identifying three margin-involved samples.

Our findings align with the previously mentioned intraoperative evaluation of breast tissues using REIMS^26,27^ and MasSpec Pen,^29^ although these studies lacked positive-margin samples for validation. Our study further supports the potential of ambient MS-based technologies in guiding surgical decisions, particularly in cases of uncertainty, thereby enabling more precise resections for improved patient outcomes. Notably, our study cohort also included patients with (18.6%) and without (81.4%) presurgical therapy, suggesting the broad applicability of the model across most breast tissue surgeries. It can potentially be applied to noncancer tissues as well, in which fibrous scarring posttherapy was also classified as tumor tissue for excision. More sample types are needed to validate the ML model’s scope of application, in addition to enhancing specificity while maintaining high sensitivity for breast surgical margin assessment.

As features with high weights are considered highly predictive of the disease and could be potential biomarkers, we proceeded to investigate the 16 nonzero weighted *m*/*z* bins in the LASSO model. The relative abundances of the 7 positively weighted and 9 negatively weighted features are shown in SI, Figure S4. Their box plots revealed trends supporting the choice of the classification features, with significant differences in their abundance among normal breast tissue and breast cancer tissue. Identification of the predictive features revealed a composition of various lipid classes, predominantly phosphatidylcholines (PC), phosphatidylethanolamine (PE) and triacylglycerols (TG) (SI, Table S2). Lipids have been reported to influence breast cancer metabolism and the tumor microenvironment, thereby affecting tumorigenesis and prognosis, making them promising markers for the disease.^29,33,36^

## Conclusions

This research offers an alternative material for swab TS-MS analysis. Furthermore, it is among the first to utilize untargeted profiles from swab TS-MS with machine learning for on-site intraoperative decision-making. The entire workflow, from sample analysis to prediction, can be completed within 5 min with high accuracy. The coupling to a compact mass spectrometer further enhances its adaptability to clinical settings. Further studies can be conducted to identify markers indicative of more aggressive breast cancers, thus requiring wider surgical margins, and to assess the applicability of this platform for classifications beyond breast cancer tissues.

## Supporting Information Available

Experimental details (including materials and methods, with additional tables and figures) (PDF)
