# Supplementary material for: Untargeted Swab Touch Spray-Mass Spectrometry Analysis with Machine Learning for On-Site Breast Surgical Margin Assessment
Source: Anal Chem. 2025 Jan 19;97(4):1960–5. doi: 10.1021/acs.analchem.4c06062 (PMC11800181; doi:10.1021/acs.analchem.4c06062)
Supplement: Supplementary file 1 — ac4c06062_si_001.pdf [file ac4c06062_si_001.pdf]

Supporting Information

Untargeted Swab Touch Spray-Mass Spectrometry Analysis with Machine Learning for On-Site Breast Surgical Margin Assessment

Laura Min Xuan Chai†, Ching Kao‡, Ming-Yang Wang‡*, Cheng-Chih Hsu†#*

† Department of Chemistry, National Taiwan University, Taipei 10617, Taiwan

‡ Department of Surgical Oncology, National Taiwan University Cancer Center, Taipei 10672, Taiwan

# Leeuwenhoek Laboratories Co. Ltd., Taipei 10672, Taiwan.

Corresponding Author

* Cheng-Chih Hsu: Email: ccrhsu@ntu.edu.tw (orcid.org/0000-0002-2892-5326

* Ming-Yang Wang: Email: A00581@ntucc.gov.tw

Table of Contents

1. Experimental Procedures and Additional References

2. Supporting Figures

3. Supporting Tables

1. Experimental Procedures

Chemicals and solvents

Methanol of LC-MS grade was purchased from Duksan Pure Chemicals (Ansan, Korea), Water of 18.2 Ω cm^{-1} resistivity was acquired using ELGA water purification system (Veolia Water Technologies, Paris, France). Acetonitrile of LC-MS grade was obtained from J.T. Baker (Phillipsburg, NJ), while LC-MS grade ethyl acetate was from Honeywell Riedel-de Haën (Seelze, Germany). ESI tuning mix was purchased from Agilent Technologies (Part No. G2421-60001).

Swabs

Omniswab was purchased from Qiagen (USA). Manufacturer describes the swab head to be made of compressed cellulose fiber, detachable from the plastic handle. Cotton swab (Brand 1) with wooden handle was purchased from Anqing Jiaxin Medical Technology (China). A swab of same material for tip and handle was purchased from Yoho Medical (Taiwan) for comparison. The polyester swab with ABS handle was purchased from Iron Will Biomedical Technology (Taiwan). Rayon and nylon flock swabs, both with PS handle were purchased from Puritan (USA).

MS raw data processing

MS raw data were exported into .cdf files and further processed using an in-house code (Spyder 5.5.1; Python 3.8.13). Briefly, the mass profile over 0.3 min was averaged. Next, *m/z* alignment was carried out, whereby the highest intensity peak over a set 1 Da region was corrected with respect to compounds with known exact mass, which were *m/z* 518.32, *m/z* 725.56, *m/z* 782.57 and *m/z* 808.58. Mass spectra were then normalized to the total ion count (TIC) and binned into 1 Da intervals.

High-weighting compound identification with liquid chromatography tandem mass spectrometry (LC-MS/MS) and their statistical significance

In our study, we utilized the compact mass spectrometer Advion Expression^{L} CMS for swab TS-MS analysis. The instrument was tuned to have a resolution of ~0.7 Da along peaks, and with this technique, simultaneous ionization of compounds occurred without separation. Hence, identification of the compounds in the *m/*z bin would require a high-resolution mass spectrometer, and coupled with liquid chromatography (LC) for added information.

For LC sample preparations, samples were first weighed (± 0.1 mg) and then extracted using a methanol, acetonitrile and ethyl acetate mixture (50:35:15, v/v/v). After adding the extraction solvent at a 1:100 sample-to-solvent ratio, the tube was sonicated for 30 minutes. The supernatant was retrieved and centrifuged at 12000 rpm for 5 min prior to analysis. The LC-MS analysis was performed using Vanquish™ system, equipped with Waters ACQUITY BEH C18 (100 × 2.1 mm, 1.7 μm) and coupled to Orbitrap Elite mass spectrometer. Mobile phase A was a mixture of acetonitrile: water (4:6, v/v), while mobile phase B had isopropanol: acetonitrile prepared at a ratio of 90:10 (v/v). Both mobile phases were added with 0.1% formic acid. The gradient begun at 25% B for 1.5 min, increasing to 55% at 3 min, followed by a gradual elution to 99% at 28.5 min, later kept at 99% B for 3 min, and returned to 25% B to condition the column for 3 min. The flow rate and column temperature were respectively set at 0.2 mL/ min and 50°C. Data were acquired in the positive-ion mode in m/z range of 100-1000. Ion source conditions were set to – heater temperature: 250°C; capillary temperature: 250°C;

source voltage: +3kV heater temperature: 250°C; sheath gas flow rate: 35 arb; auxiliary gas flow rate: 30 arb. Mass spectrometer was operated in FT mode at 30,000 resolution. The HCD fragmentation was investigated at 25, 30, 35 and 40 arb, with an isolation window of 1.5 Da.

Putative compound identification was then assigned by referring to its exact mass (mass error within 5 ppm), and fragmentation pattern of the tandem mass spectrum. Compounds were assigned by cross-referencing with the databases, METLIN[1] and LIPID MAPS.[2,3] As each *m/*z bin may contain several compounds, the compound with the highest abundance in the sample group that the *m/z* bin is predictive of was identified as the representative compound. For example, *m/z* bin 764.5 is negatively-weighted, thus potentially a biomarker of normal breast tissue. The base peak chromatogram from *m/*z 764.0-765.0 revealed that *m/*z 764.6759 at RT 23.90 min had highest abundance in normal breast tissue, therefore was selected as the representative compound of the *m/z* bin. In situations where there are multiple highly-intensity compounds in the *m/z* bin, all potential compounds were identified after examining the change in abundance between sample groups.

To determine the statistical significance of the high-weight features, Welch t-test was then performed on normal breast tissue and breast cancer tissue swabs using SciPy package and box plots were generated with matplotlib package. Note that the patient with fibrous scarring post therapy was not included in the plot.

References

(1) Smith, C. A.; Want, E. J.; O'Maille, G.; Abagyan, R.; Siuzdak, G. METLIN: A Metabolite Mass Spectral Database. *Therapeutic Drug Monitoring.* **2005**, *27* (6), 747–751.

(2) Sud, M.; Fahy, E.; Cotter, D.; Brown, A.; Dennis, E.; Glass, C.; Murphy, R.; Raetz, C.; Russell, D.; Subramaniam, S. LMSD: LIPID MAPS Structure Database. *Nucleic Acids Research.* **2006**, *34* (suppl. 1), D527–D532.

(3) Conroy, M. J.; Andrews, R. M.; Andrews, S.; Cockayne, L.; Dennis, E. A.; Fahy, E.; Gaud, C.; Griffiths, W. J.; Jukes, G.; Kolchin, M.; Mendivelso, K.; Lopez-Clavijo, A. F.; Ready, C.; Subramaniam, S.; O'Donnell, V. B. LIPID MAPS: Update to Databases and Tools for the Lipidomics Community. *Nucleic Acids Research.* **2023**, *51* (D1), D780–D787.

2. Supporting Figures

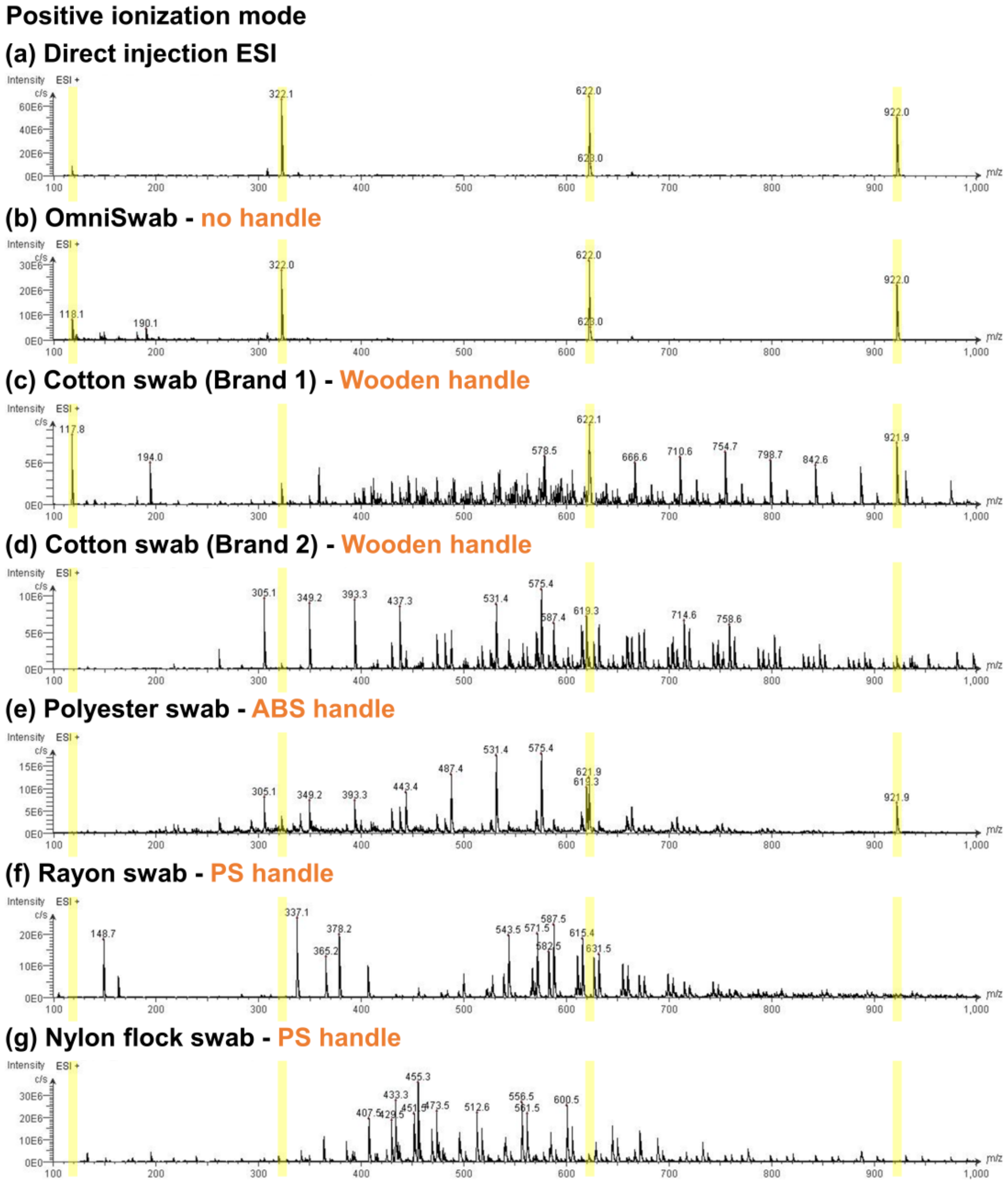

Figure S1. Comparison of the background of different swab materials in positive ionization mode. ESI tuning mix with *m/z* peaks of 118.09, 322.05, 622.03 and 922.01 acted as references during swab TS-MS analysis.

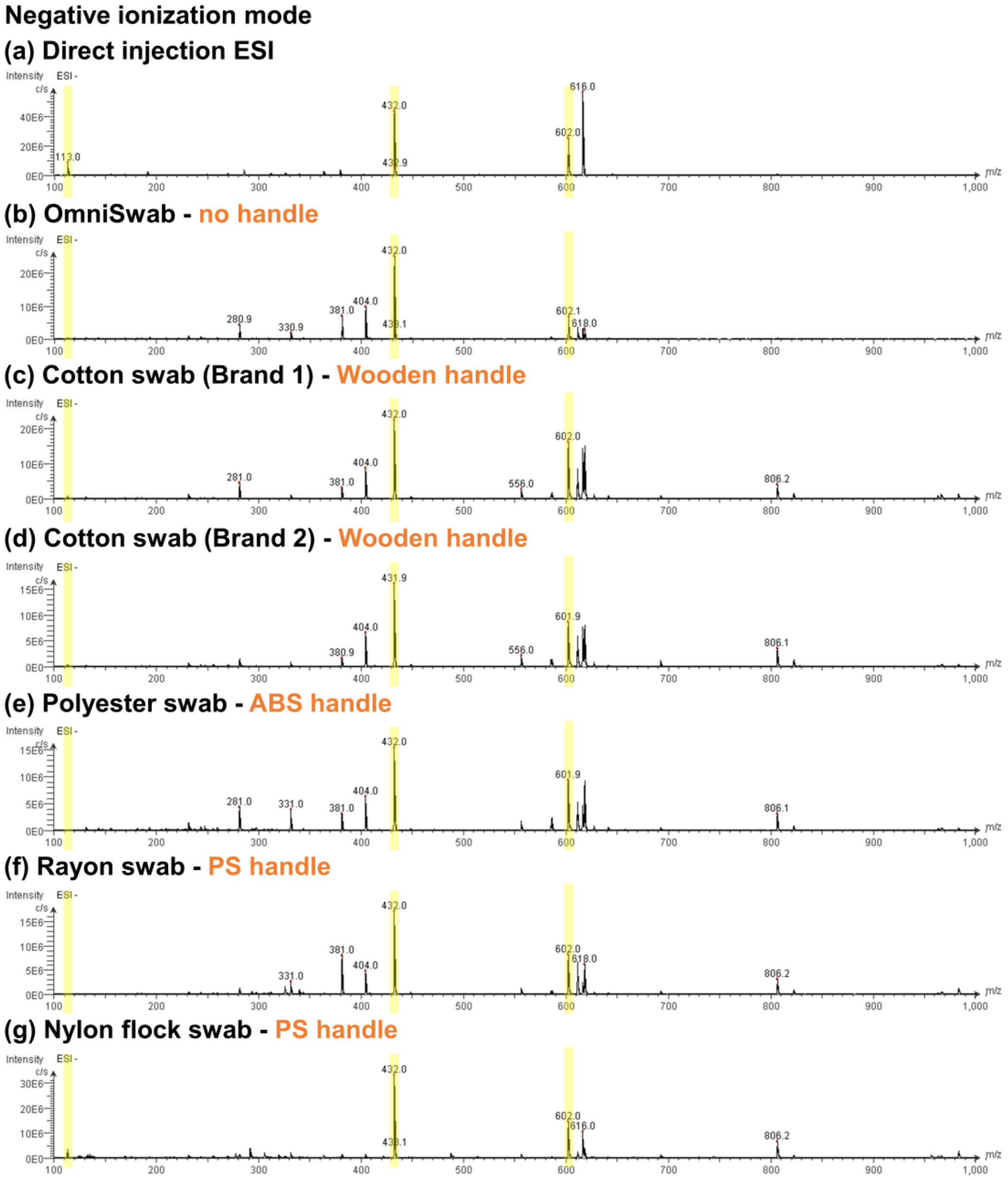

Figure S2. Comparison of the background of different swab materials in negative ionization mode. ESI tuning mix with *m/z* peaks of 112.99, 431.98 and 601.98 acted as references during swab TS-MS analysis.

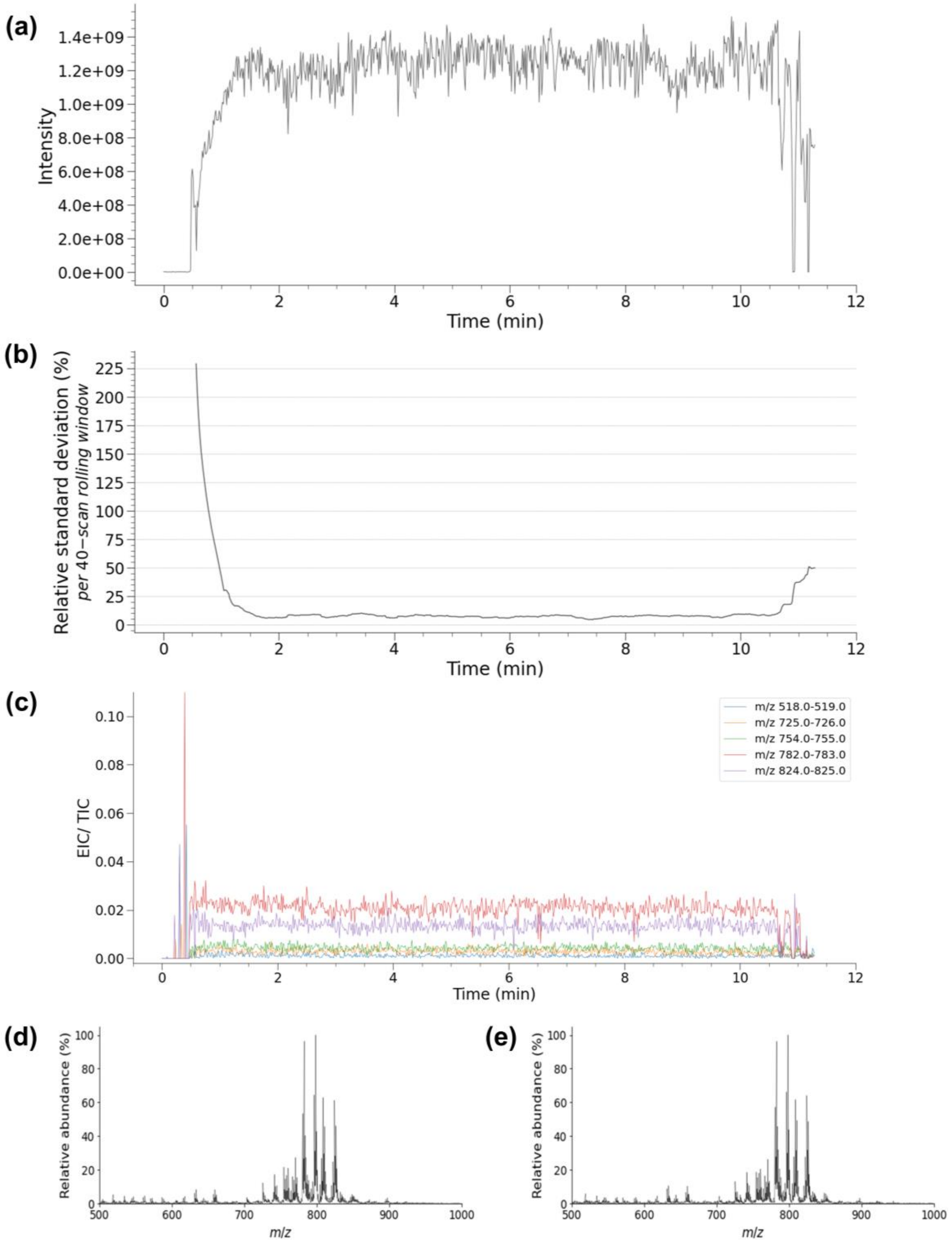

Figure S3. A breast surgical margin swab analyzed by swab TS-MS. **(a)** Total ion count (TIC) chromatogram and **(b)** relative standard deviation of a 40-scan (~0.3 min) rolling window, showing changes in TIC during swab TS-MS analysis. Briefly, a high voltage of 4 kV was applied, and mass acquisition begun at 0 min. Rinse solvent was added at 0.4 min, and the continuous extraction solvent delivery, directed towards the top corner of the swabbing edge, begun at 0.5 min. A droplet was observed at the apex of the swab tip, allowing Taylor cone formation. TIC signals stabilized at 1.2 min, with RSD below 15%. Gradual enlargement of suspended droplet was observed, where sharp drops in intensities occurred after 10.7 min due to Taylor cone pulsations. **(c)** The extracted ion count (EIC) chromatogram of 5 *m/z* compounds (1-Da interval) was normalized by TIC, showing stable normalized intensities at 0.7 min, despite TIC still exhibiting an increasing trend. Mass profiles averaged over 0.3 min at different times – **(d)** 2.0 to 2.3 min, and **(e)** 5.0 to 5.3 min – were similar.

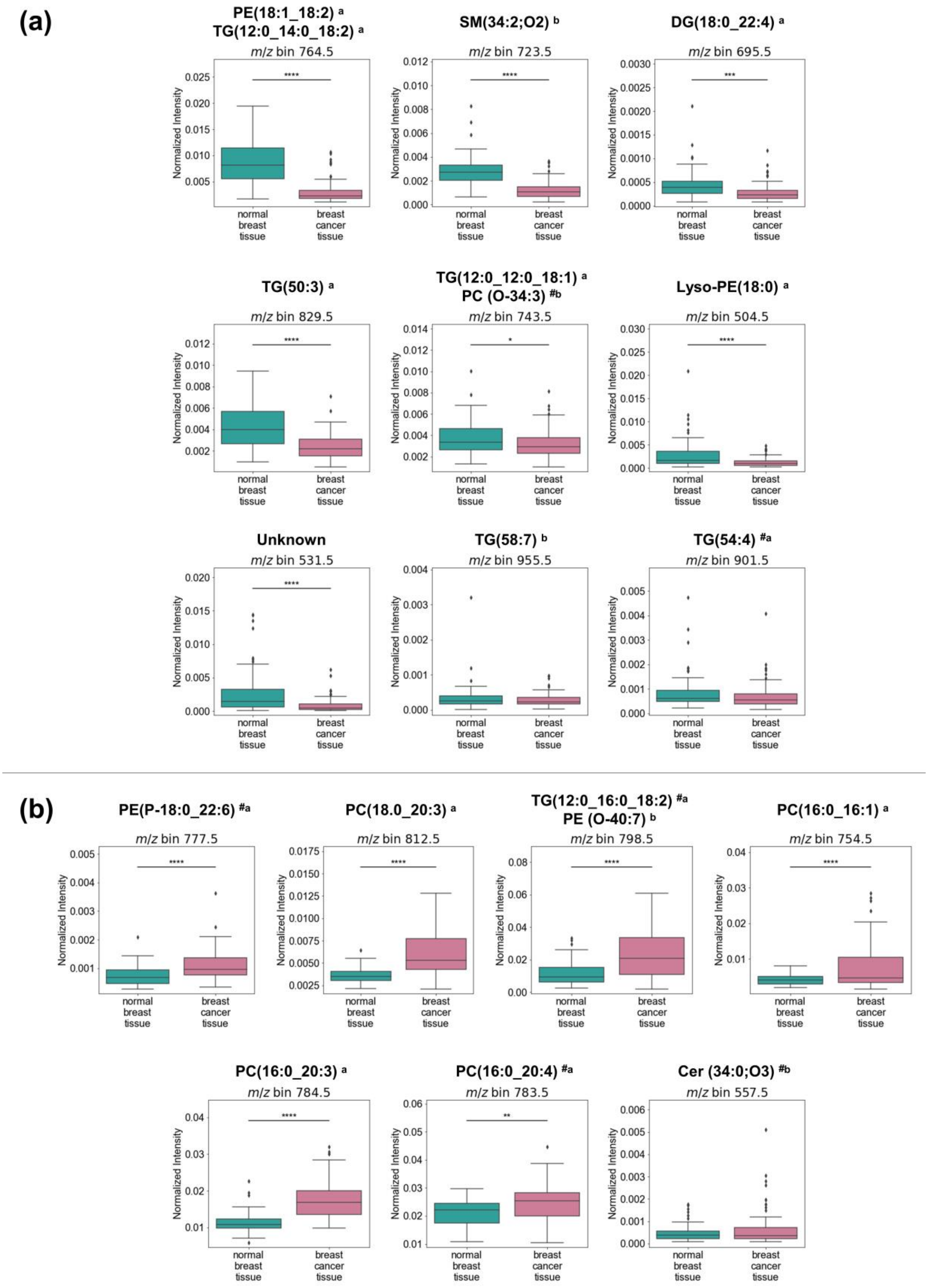

Figure S4. Boxplot of the **(a)** negatively-weighted and **(b)** positively-weighted *m/z* bins in the LASSO model, showing normalized intensities in normal breast tissue and breast cancer tissue. Statistical significance is indicated by asterisks (**p*<0.05, ***p*<0.01, ****p*<0.001, *****p*<0.0001). *Abbreviations*: phosphatidylethanolamine (PE); phosphatidylcholines (PC); triacylglycerol (TG); ceramide (Cer); sphingomyelin (SM); diacylglycerol (DG); [#]: isotope peak; [a]: putative ID of compounds assigned by referring to its exact mass (mass error within 5 ppm), and fragmentation pattern of the tandem mass spectrum; [b]: putative ID of compounds assigned by referring to its exact mass (mass error within 5 ppm).

3. Supporting Table

Tables S1. Patient demographics and sample pathology results

**No. of patients**	97
**Age, median (IQR), year**	57 (48-65)
	**No. (%)**
**Gender**	
Female	97 (100)
**No. of Samples**	
Normal Breast Tissue	74 (46.3)
No presurgical therapy	*59 (79.7)*
Post-neoadjuvant therapy	*15 (20.3)*
Breast Tumor	86 (53.7)
No presurgical therapy	*74 (86.0)*
invasive carcinoma of no special type	44 (59.5)
carcinoma in situ	10 (13.5)
invasive lobular carcinoma	6 (8.1)
mucinous carcinoma	5 (6.8)
mixed carcinoma	4 (5.4)
metaplastic carcinoma	1 (1.3)
invasive carcinoma of apocrine features	1 (1.3)
margin involved with invasive carcinoma	2 (2.7)
margin involved with DCIS	1 (1.3)
Post-neoadjuvant therapy	*12 (14.0)*
invasive carcinoma of no special type	9 (75.0)
mixed carcinoma	1 (8.3)
fibrosis	1 (8.3)
margin involved with invasive carcinoma	1 (8.3)

Tables S2. Identification of high-weighted features selected by LASSO model.

**Features predictive of breast cancer tissue**				**Features predictive of normal tissue**			
***m/z* bin**	**Weight**	**Detected exact mass**	**Proposed identification**	***m/z* bin**	**Weight**	**Detected exact mass**	**Proposed identification**
777.5	0.275	777.5611	PE(P-18:0_22:6) [#a]	764.5	-0.595	764.6759	TG(12:0_14:0_18:2) [a]
						764.5197	PE(18:1_18:2) [a]
812.5	0.213	812.6156	PC(18.0_20:3) [a]	723.5	-0.533	723.5416	SM(34:2;O2) [b]
798.5	0.184	798.6652	TG(12:0_16:0_18:2) [#a]	695.5	-0.151	695.5572	DG(18:0_22:4) [a]
		798.5393	PE (O-40:7) [b]				
754.5	0.164	754.5344	PC(16:0_16:1) [a]	829.5	-0.107	829.7239	TG(50:3) [a]
784.5	0.060	784.5844	PC(16:0_20:3) [a]	743.5	-0.098	743.6155	TG(12:0_12:0_18:1) [a]
						743.5405	PC (O-34:3) [#b]
783.5	0.058	783.5717	PC(16:0_20:4) [#a]	504.5	-0.088	504.3059	Lyso-PE(18:0) [a]
557.5	0.020	557.5326	Cer (34:0;O3) [#b]	531.5	-0.040	531.4851	-
				955.5	-0.018	955.7680	TG(58:7) [b]
				901.5	-0.008	901.8033	TG(54:4) [#a]

[#] : isotope peak;

[a] : putative ID of compounds assigned by referring to its exact mass (mass error within 5 ppm), and fragmentation pattern of the tandem mass spectrum;

[b] : putative ID of compounds assigned by referring to its exact mass (mass error within 5 ppm).

Abbreviations: phosphatidylethanolamine (PE); phosphatidylcholines (PC); triacylglycerol (TG); ceramide (Cer); sphingomyelin (SM); diacylglycerol (DG)
